# Progesterone inhibits contraction and increases TREK-1 potassium channel expression in late pregnant rat uterus

**DOI:** 10.18632/oncotarget.23084

**Published:** 2017-12-07

**Authors:** Zongzhi Yin, Yun Li, Wenzhu He, Dan Li, Hongyan Li, Yuanyuan Yang, Bing Shen, Xi Wang, Yunxia Cao, Raouf A. Khalil

**Affiliations:** ^1^ Department of Obstetrics and Gynecology, The First Affiliated Hospital of Anhui Medical University, Hefei, China; ^2^ Reproductive Medicine Center, Department of Obstetrics and Gynecology, The First Affiliated Hospital of Anhui Medical University, Hefei, China; ^3^ Anhui Province Key Laboratory of Reproductive Health and Genetics, Anhui Medical University, Hefei, China; ^4^ Anhui Provincial Engineering Technology Research Center for Biopreservation and Artificial Organs, Hefei, China; ^5^ Department of Scientific Research, The Second Affiliated Hospital of Anhui Medical University, Hefei, China; ^6^ Department of Physiology, Anhui Medical University, Hefei, China; ^7^ Vascular Surgery Research Laboratories, Division of Vascular and Endovascular Surgery, Brigham and Women’s Hospital, Harvard Medical School, Boston, MA, USA

**Keywords:** pregnancy, uterine contraction, progesterone, TREK-1 channel

## Abstract

**Objective:**

The aim of this study was to investigate the effect and mechanism by which progesterone regulates uterine contraction in late pregnant rats

**Results:**

Progesterone caused concentration-dependent relaxation of uterine strips that was enhanced compared with control nontreated uterine strips. Uterine strips incubated with progesterone showed a significant increase in TREK-1 mRNA expression and protein level. TREK-1 inhibitor L-methionine partly reversed uterine relaxation caused by the progesterone, while TREK-1 activator arachidonic acid did not cause significant change in progesterone-induced relaxation.

**Conclusions:**

Progesterone inhibits uterine contraction and induces uterine relaxation in late pregnancy. The progesterone-induced inhibition of uterine contraction appears to partly involve increased potassium channel TREK-1 expression/activity.

**Materials and Methods:**

Uterus from late-pregnant rats (gestational day 19) was isolated, and uterine strips were prepared for isometric contraction measurement. Oxytocin-induced contraction was compared in uterine strips pretreated with different concentration of progesterone. TREK-1 potassium channel inhibitor L-methionine and TREK-1 agonist arachidonic acid were used to determine whether the changes caused by progesterone involve changes in TREK-1 activity. The mRNA and protein expression of TREK-1 in uterine tissues were measured using qPCR and Western blot.

## INTRODUCTION

During the course of normal pregnancy, the human uterus expands in volume dramatically due to hypertrophy and distension of uterine smooth muscle, thus allowing sufficient space and nutrition for the developing fetus [1]. In contrast, the uterus is like a sleeping giant during the third trimester. Once the uterus is awaken during full-term, it transforms into an excitable state and becomes one of the strongest muscles in the human body in order to facilitate birth [2, 3]. However, the mechanisms underlying uterine quiescence during pregnancy and its remarkable transformation and excitability at term remain major unanswered questions for obstetricians [4].

Pregnancy is a physiological process encompassing significant hormone-mediated adjustments. One of the most pivotal players during pregnancy is the steroid hormone progesterone [5]. Clinical trials have shown beneficial effects of supplementation with progesterone in preventing preterm labor in women at risk for preterm delivery [6–8]. It is now generally recognized that progesterone is essential for maintenance of pregnancy and promotes uterine relaxation during early pregnancy, and its functional withdrawal may initiate parturition [9, 10]. However, the mechanisms via which progesterone regulates uterine contraction are not fully understood.

Serval potassium channel subtypes have been suggested to affect uterine function, and to facilitate uterine quiescence to full term during pregnancy [11]. TREK-1 channel, a stretch-activated tetraethyl ammonium-insensitive potassium (K^+^) channel, has been described in smooth muscle of human myometrium. TREK-1 decreases myometrial smooth muscle excitability by helping to return depolarized cells to a more negative resting potential and to maintain resting cells near the K^+^ equilibrium potential [12, 13]. Researches have shown that TREK-1 expression is reduced in full-term and preterm laboring compared to non-laboring human myometrium, an observation consistent with a role for TREK-1 in maintaining quiescence during pregnancy [14]. This led to the suggestion that TREK-1 up-regulation could potentially contribute to maintenance of uterine quiescence, while its down-regulation could lead to critical transition from quiescent to laboring myometrium.

Although previous studies have shown that progesterone causes relaxation of uterine smooth muscle, and TREK-1 channel may have regulatory effects related to maintenance of myometrium quiescence, the interrelationship between progesterone and TREK-1 channel function in uterine relaxation during late pregnancy is unclear. The present study sought to investigate the role of TREK-1 channel in progesterone-induced uterine relaxation. We used the late pregnant rat uterus to investigate whether: (1) Progesterone inhibits uterine contraction during late pregnancy, (2) The TREK-1 channel contributes to progesterone-induced regulation of uterine contraction.

## RESULTS

### Progesterone dose-dependently inhibits contraction of late pregnant uterus

**Figure 1:**
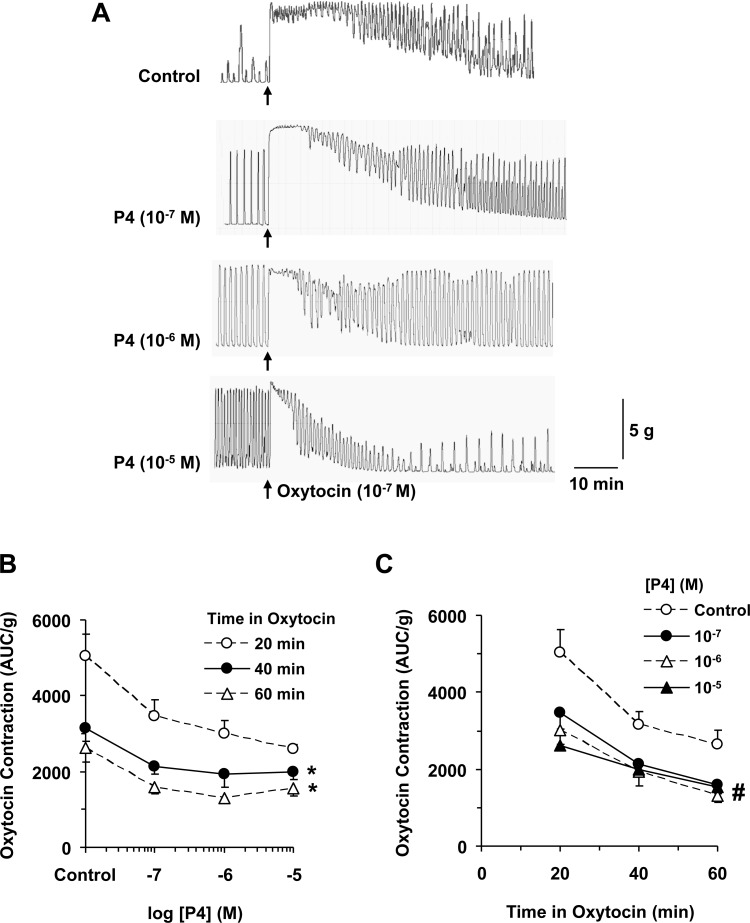
Effects of progesterone on oxytocin-induced contraction Uterine strips of late-pregnant rats were either nontreated (control) or pretreated with increasing concentrations of progesterone (P4, 10^–7^ to 10^–5^ M) then stimulated with oxytocin (10^–7^ M) and the contractile response was recorded (**A**). The AUC/g tissue weight of oxytocin-induced contraction was calculated for 20, 40, 60 min, and presented for each time period at different concentrations of P4 pretreatment (**B**), or for each P4 concentration at different time periods (**C**). Data represent means ± SEM, *n* = 5–10. ^*^Significantly different (*P* > 0.05) from measurements after 20 min. ^#^Significantly different (*P* < 0.05) from control tissues nontreated with P4.

Uterine strips of late pregnant rats showed significant contraction to oxytocin. Oxytocin is a neuropeptide hormone and a nonapeptide with a relatively short half life (∼15 min), and therefore many traditional protocols for oxytocin infusion regimens in humans recommend increases of infusion rate at 15–20 min intervals. However, recent clinical studies suggest that prolonged intervals of 30–40 or even 60 min could be superior to shorter dosage intervals in terms of efficacy and safety, and that increasing oxytocin infusion earlier could cause excessive uterine contraction and fetal distress. Given the pharmacological data on oxytocin in pregnant women which show a half-life of ∼15 min, and the pharmacokinetic principle that at least 3 half-lives of constant infusion may elapse before the corresponding clinical effect is established [15], it would be important to investigate the effects of oxytocin for extended time periods between 40 to 60 min. It should also be noted that the time course of the effects of oxytocin is relatively shorter *in vivo* because of the presence of endogenous metabolizing and degrading enzymes. In comparison, the time course of the effects of oxytocin is expected to be longer in the present *ex vivo* experiments in the absence of metabolizing enzymes. Our present control experiments showed that oxytocin induced an initial mainly tonic response for 5 to 10 min, followed by both tonic and phasic contractile response that showed some decline over a period of 20, 40 and 60 min (Figure 1).

We tested the effects of pretreatment of uterine strips with progesterone on oxytocin-induced contraction. Uterine strips were incubated under 2 g tension for 1 h in the presence of progesterone (10^–7^ to 10^–5^ M) (Figure 1A). The data from control nontreated tissues and tissues treated with progesterone were analyzed similarly, and therefore any decline in oxytocin activity that occurred in the control strips was factored in the calculation. In comparison with control tissues, progesterone pretreated tissues showed further reduction in oxytocin-induced contraction, with the contractile response measured after 40 and 60 min significantly reduced compared to the contractile response measured after 20 min (Figure 1B). Also, the reduction in oxytocin contraction was dependent on progesterone concentration, with the contractile response significantly reduced with increasing concentrations of progesterone compared to control tissues without progesterone treatment (Figure 1C).

### TREK-1 mRNA and protein amount increased in progesterone pretreated late-pregnant uterus

**Figure 2:**
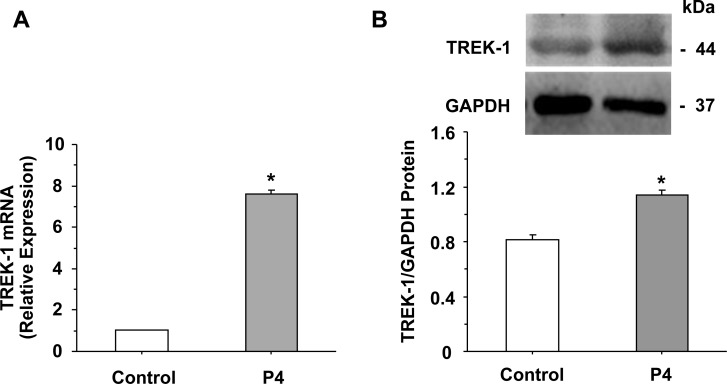
TREK-1 mRNA expression and protein level Uterine strips from late-pregnant uterus were either nontreated (control) or pretreated with progesterone (P4, 10^–5^ M) for 1 h. Tissues were prepared for qPCR, and the mRNA expression of TREK-1 channel was measured relative to the housekeeping gene GAPDH (**A**). Uterine strips were also homogenized in preparation for Western blot analysis, and the optical density of TREK-1 was normalized to GAPDH (**B**). Data represent means ± SEM, *n* = 7. ^*^Significantly different (*P* < 0.05), P4 vs control.

Q-PCR analysis revealed expression of TREK-1 mRNA in uterine strips of late-pregnant rats. TREK-1 mRNA expression was significantly enhanced in uterine strips treated with progesterone compared with control non-treated uterine strips (Figure 2A).

Western blot analysis revealed a prominent band at 44 kDa corresponding to TREK-1 in uterine strips of late-pregnant rats. The protein amount of TREK-1 was significantly enhanced in uterine strips treated with progesterone compared with control non-treated uterine strips (Figure 2B).

### TREK-1 inhibitor L-methionine reverses progesterone-induced relaxation of late-pregnant uterus

**Figure 3:**
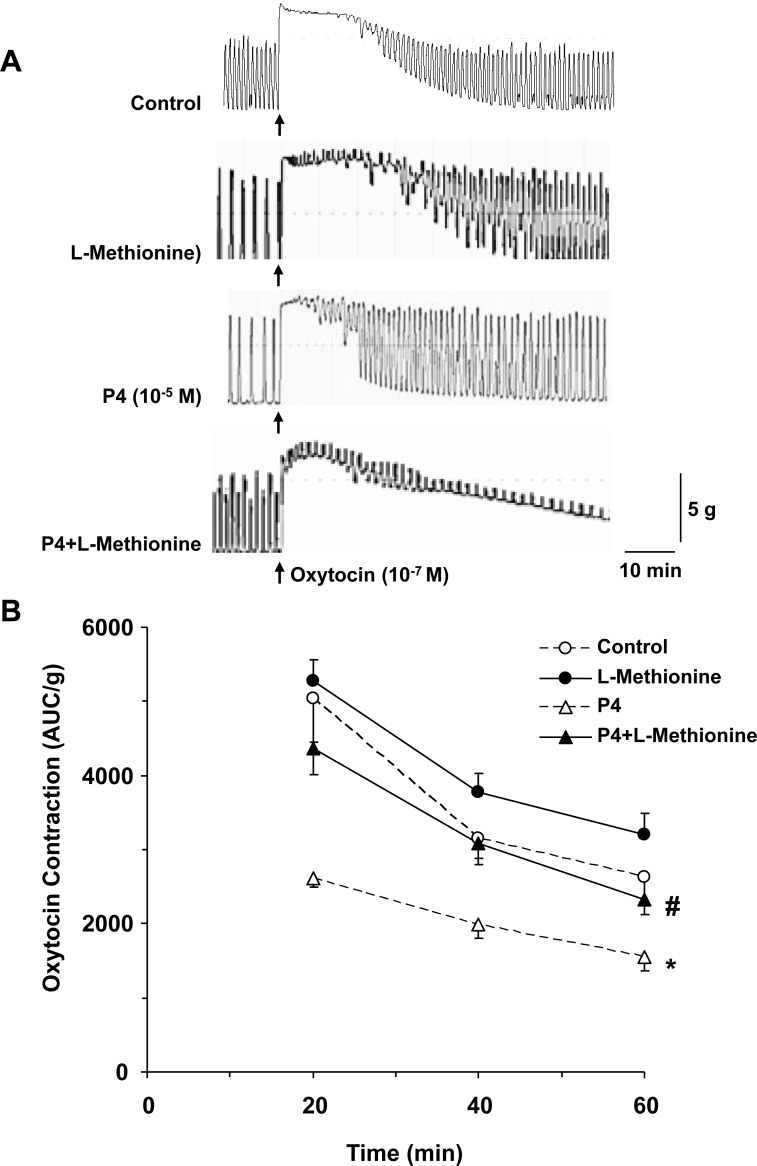
Effect of inhibition of TREK-1 channel activity on uterine contraction Uterine strips from late-pregnant were either nontreated (control), or pretreated with TREK-1 inhibitor L-methionine (1 mM), progesterone (P4, 10^–5^ M), or P4+L-methionine for 1 h then stimulated with oxytocin (10^–7^ M) for 1 h (**A**). Oxytocin-induced contraction in AUC/g tissue weight was calculated for 20, 40, and 60 min (**B**). Data represent means ± SEM, *n* = 5–10. ^*^Significantly different (*P* < 0.05) from control nontreated tissues. ^#^Measurement in tissues pretreated with P4+L-methionine are significantly different (*P* < 0.05) from tissues pretreated with P4 alone.

To determine whether the progesterone-induced changes in uterine contraction are related TREK-1 activity, we tested the effects of TREK-1 inhibitor L-methionine (1 mM) (Figure 3A). Uterine strips under 2 g basal tension and treated with L-methionine for 1 h showed an insignificant enhancement of uterine contraction compared to control tissues without L-methionine treatment. Importantly, in uterine strips treated simultaneously with progesterone and L-methionine, no significant reduction in uterine contraction was observed, and the uterine contraction was at levels similar to those in control tissues without progesterone or L-methionine treatment, suggesting opposing effects of L-methionine and progesterone on TREK-1 channel-mediated uterine relaxation (Figure 3B)

### TREK-1 activator arachidonic acid causes no further effect on progesterone-induced uterine relaxation

**Figure 4:**
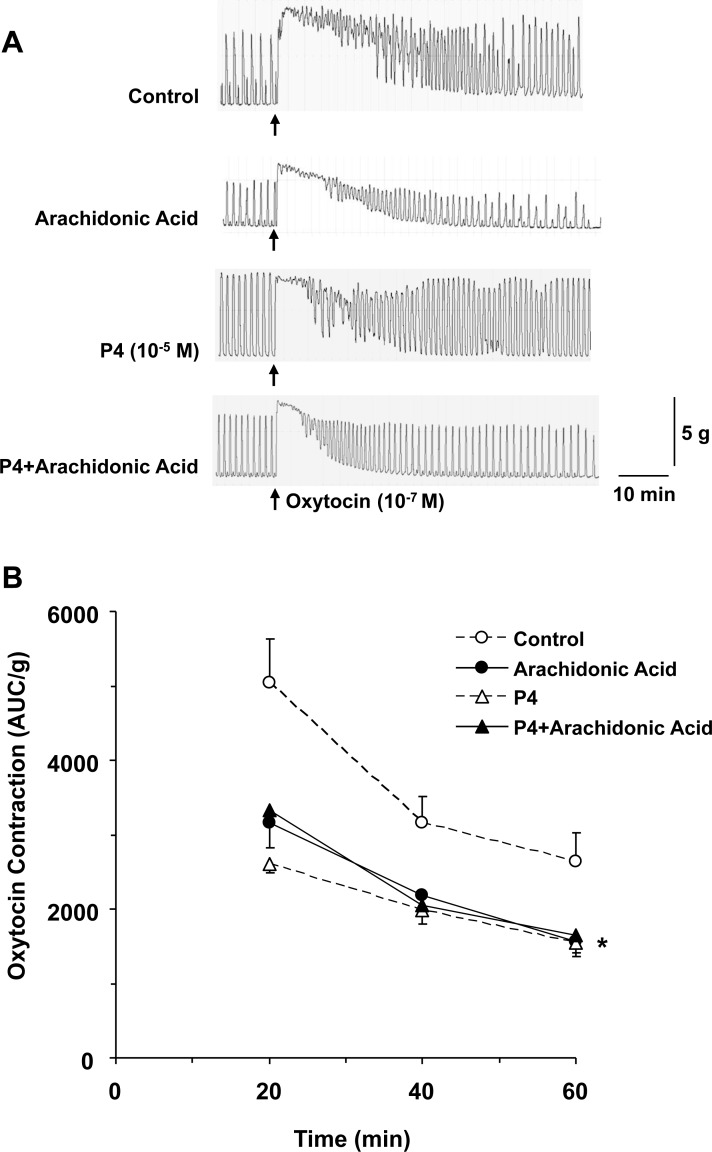
Effect of activation of TREK-1 channel on uterine contraction Uterine strips from late-pregnant were either nontreated (control), or pretreated with TREK-1 activator arachidonic acid (10^–5^ M), progesterone (P4, 10^–5^ M), or P4+arachidonic acid for 1 h then stimulated with oxytocin (10^–7^ M) for 1 h (**A**). Oxytocin-induced contraction in AUC/g tissue weight was calculated for 20, 40, and 60 min (**B**). Data represent means±SEM, *n* = 5–10. ^*^Significantly different (*P* < 0.05) from control nontreated tissues.

We tested the effects of TERK-1 activator arachidonic acid on oxytocin-induced uterine contraction both in the absence and presence of progesterone (Figure 4A). In uterine strips pretreated with arachidonic acid (10^–5^ M) for 1 h, oxytocin contraction was significantly reduced to with control tissues without arachidonic acid treatment. No statistical difference in oxytocin contraction was observed in tissues treated with progesterone plus arachidonic acid compared with the uterine strips treated with arachidonic acid alone or progesterone alone (Figure 4B), suggesting that both arachidonic acid and progesterone activate TREK-1 mediated uterine relaxation to a similar extent

### Uterine stretch causes no further effect on progesterone-induced uterine relaxation

**Figure 5:**
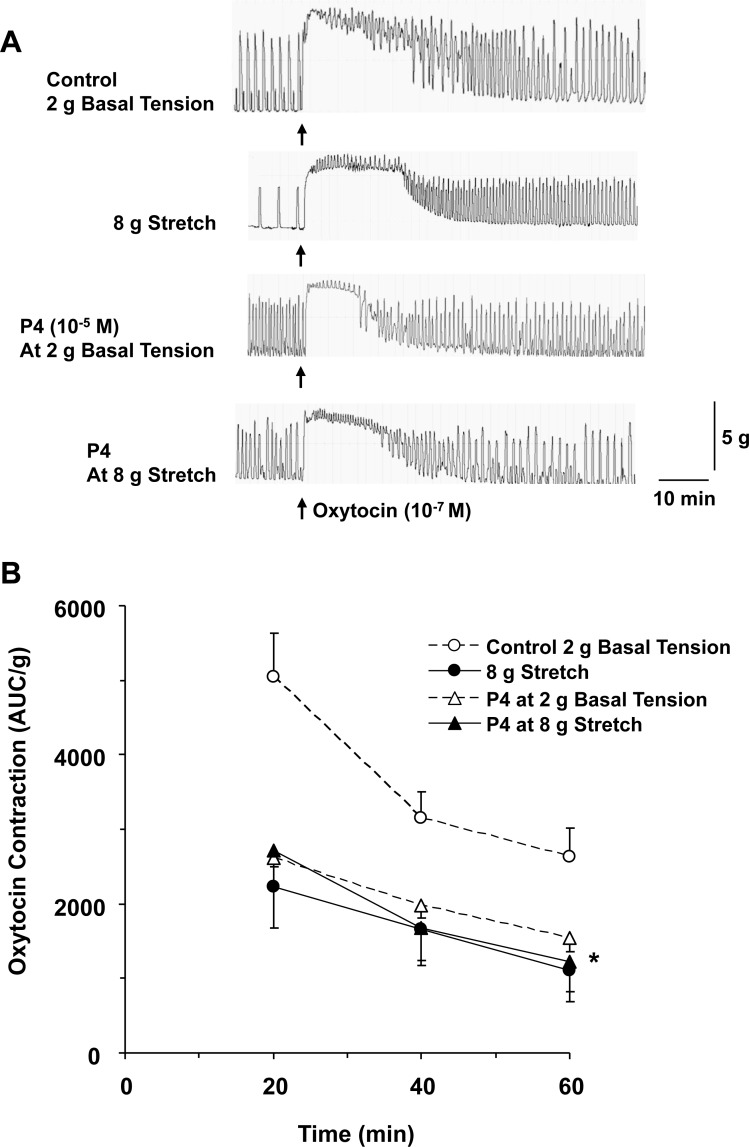
Effects of prolonged stretch and progesterone on oxytocin contraction Uterine strips from late-pregnant were equilibrated either under 2 g basal tension and nontreated (control), under 2 g basal tension and pretreated with progesterone (P4, 10^–5^ M), under 8 g stretch and nontreated, or under 8 stretch and pretreated with P4 for 1 h then stimulated with oxytocin (10^–7^ M) for 1 h (**A**). Oxytocin-induced contraction in AUC/g tissue weight was calculated for 20, 40, and 60 min (**B**). Data represent means ± SEM, *n* = 5–10. ^*^Significantly different (*P* < 0.05) from control nontreated tissues under 2 g basal tension.

In our previous study, we found that both normal pregnancy and prolonged uterine stretch are associated with decreased myometrium contraction [1]. We studied the combined effect of prolonged stretch in association with progesterone on contraction of late pregnant uterus (Figure 5A). Uterine strips were incubated under 2 g basal tension or 8 g stretch for 1 h in the absence or presence of progesterone (10^–5^ M), then stimulated with oxytocin (10^–7^ M). Uterine stretch under 8 g tension caused reduction in oxytocin-induced contraction. However, the reduction in uterine contraction in tissues under 8 g stretch tension and pretreated with progesterone was not statistically different from tissues incubated under 8 g stretch alone, or in tissues under 2 g basal tension and pretreated with progesterone (Figure 5B)

## DISCUSSION

The present study shows that progesterone inhibits contraction in late pregnant rat uterus, and that the contraction inhibition is partly related to increased TREK-1 expression and activity during late pregnancy.

During normal pregnancy, the balance between uterine contraction and relaxation is tightly regulated in order to maintain healthy and full-term pregnancy. Changes in the uterine relaxation mechanisms could cause unexpected uterine contraction with untoward outcomes to the pregnancy, and could result in a premature and underdeveloped newborn. However, the mechanisms that regulate myometrium excitability and contraction during pregnancy and at the onset of labor are not fully understood.

Progesterone has been used to inhibit uterine contraction and prevent abortion and preterm labor. Our study demonstrates that progesterone inhibits uterine contraction in late pregnant rats. These observations are consistent with the myometrium relaxing effects of progesterone in the early stages of pregnancy. Our results are also consistent with clinical studies demonstrating beneficial effects of acute administration of progesterone at term [16]. However, the mechanisms underlying progesterone-induced inhibition of myometrium contraction are not clearly understood.

Studies have related the relaxing effects of progesterone on the myometrium to both genomic and non-genomic actions mediated by progesterone receptors [17]. Progesterone-activated genomic pathways could limit the expression of contractile agonists (e.g. oxytocin), receptors (e.g. prostaglandin receptors), contraction-associated protein (e.g. gap junctions), or microRNAs (e.g. miR-200 family) within the myometrium, and in turn cause long-term modulation of the uterine contractile phenotype [5, 18–20]. On the other hand, progesterone-activated non-genomic pathways are more rapid and could affect the contractile machinery by modulating intracellular signal transduction pathways [17], including ion channels [21].

K^+^ channels are important regulators of membrane potential, tissue excitability and smooth muscle contraction. Several types of K^+^ channels have been identified in different tissues and cells, including small conductance Ca^2+^-activated K^+^ channels (SK_Ca_), intermediate conductance Ca^2+^-activated (IK_Ca_), large conductance Ca^2+^- and voltage-activated (BK_Ca_), voltage-dépendent (K_V_), ATP-sensitive (K_ATP_) K^+^ channels, and the inward rectifier (K_ir_) [22–26]. The TREK-1 channel belongs to two-pore K^+^ channels (K_2P_), which are largely expressed in visceral smooth muscle [12]. TREK-1 channels are expressed in human myometrium particularly during pregnancy [14, 27], and are thought to maintain background outward K^+^ current and resting membrane potential and thereby counterbalance membrane depolarization and muscle contraction [28–30]. TREK-1 channel activity has been shown to be regulated by numerous factors, including pH, temperature, phosphorylation, nitric oxide [14]. TREK-1 activity can also be modulated by pharmacological agents such as arachidonic acid and L-methionine, as well as mechanical tissue stretch [31]. Consistent with studies in the human uterus, the present qPCR and Western blot analyses demonstrated detectable amounts of TREK-1 mRNA and protein in late pregnant rat uterus. The present study showed that treatment of uterine strips with TREK-1 channel inhibitor L-methionine insignificantly increased uterine contraction, while treatment with TREK-1 activator arachidonic acid caused a dramatic reduction in uterine contraction, suggesting that the TREK-1 is active and functional during pregnancy in rats.

The present study suggests both genomic and nongenomic effects of progesterone on uterine TREK-1 expression/activity. Our qPCR and western blot analyses showed that treatment of uterine strips with progesterone increased TREK-1 mRNA expression and protein level. To test whether progesterone inhibits myometrial contractility in late-pregnant rats by affecting TREK-1 channels function, we measured the effects of progesterone on oxytocin-induced uterine contraction in the presence of TREK-1 modulators. We found that the progesterone-induced inhibition of uterine contraction was reversed in the presence of the TREK-1 inhibitor L-methionine, suggesting that progesterone and L-methionine could have opposing effects on TREK-1 activity. On the other hand, progesterone did not cause any further inhibition of uterine contraction in tissues pretreated with TREK-1 activator arachidonic acid, suggesting that progesterone and arachidonic acid may have similar effects on TREK-1 activity. The restricted effects of progesterone on uterine relaxation in tissues treated with arachidonic acid are most likely due to the fact that the TREK-1 channel has limitation in maximum activation. Taken together these observations suggest that TREK-1 function plays a pivotal role in the progesterone-induced regulation of uterine contraction in late pregnancy.

In a previous study, we have shown that prolonged stretch during pregnancy is associated with increased expression and activity of specific matrix metalloproteinases (MMPs), which could in turn inhibit uterine contraction and promote uterine relaxation [1]. MMPs are largely known for their role in tissue remodeling and degradation of extracellular matrix proteins [32, 33]. We and others have recently shown other novel effects of MMPs on cell surface receptors and signaling molecules [33, 34]. Specifically, we have shown that MMP-2 can induce relaxation of rat inferior vena cava via hyperpolarization and activation of large conductance Ca^2+^-activated K^+^ channels [35]. These observations suggest that uterine stretch could activate K^+^ channels and promote uterine relaxation though increased expression/activity of MMPs. Interestingly, TREK-1 channels are also sensitive to mechanical stimuli [31]. Consistent with our previous report [1], the present study showed that prolonged stretch caused a decline in oxytocin-induced uterine contraction. Importantly, progesterone did not cause any further inhibition of uterine contraction in tissues under prolonged and excessive stretch, supporting that both progesterone and stretch could cause uterine relaxation via a similar pathway. Assuming that both progesterone and stretch act via activation of K^+^ channels, then the lack of effects of progesterone in stretch uterine tissues could be due to the fact that K^+^ channels have limitations in their maximal activation.

In human pregnancy, progesterone administration is considered an efficient approach to prevent preterm birth in the third trimester by promoting myometrial relaxation. The present study was performed on isolated uterus from pregnant rats, and species differences should be considered when interpreting the data. Also, the present study used progesterone at relatively high concentration, and future experiments should test both the acute and long-term effects of more physiological concentrations of progesterone. Given that the sex hormone balance in late pregnancy is extremely complex; the changes in progesterone concentration represent only a small part of the multiple hormonal changes that could be more apparent in *in vivo* studies. The present study also examined the effects of progesterone on the expression/activity of TREK-1 channel in late pregnant rats on gestational day 19–20. Future studies should examine the time course of the changes in expression/activity of TREK channel, and specifically determine whether these changes are reversible with the physiological decline in progesterone levels on gestational day 22 and immediately prior to the onset of labor in rats. Also, while the present data suggest possible role of progesterone on TREK-1 channel expression/activity, other effects of progesterone on mechanisms of uterine contraction can not be excluded, and these limitations should be addressed and further examined in future studies.

In summary, the present study demonstrates that progesterone can maintain the balance of uterine contraction and relaxation in late-pregnant rats. TREK-1 channel appears to play a role in regulating uterine contraction, and progesterone-induced uterine relaxation appears to partly involve increased uterine TREK-1 expression/activity. Future experimental and clinical studies should test whether TREK-1 activators would be useful in combination with progesterone to prevent preterm birth, and whether TREK-1 inhibitors can be useful perhaps in combination with oxytocin to initiate labor.

## MATERIALS AND METHODS

### Tissue preparation

The study was conducted on late-pregnant Sprague-Dawley rats (Gestational day 19–20, 12 weeks of age, 300 to 350 gram (g) weight). The rats were housed in the animal facility and maintained on ad libitum standard rat chow and tap water in 12:12-h light-dark cycle. Pregnant rats were euthanized using two methods of euthanasia comprising inhalation of CO_2_ until complete cessation of breathing and heart beats, followed by a second method of euthanasia comprising exsanguination, bilateral pneumothorax and tissue harvest. All procedures were performed in accordance with the guidelines of the Institutional Animal Care and Use Ethics Committee at the First Affiliated Hospital of Anhui Medical University, Hefei, China.

The uterus of pregnant rats was rapidly excised and the fetuses and placentae were removed. The uterus was immersed in Krebs solution and the fat and connective tissue were cleaned under microscopic visualization. From each rat, longitudinal uterine strips (3 mm × 7 mm, width × length) were isolated and suspended vertically in four individual, temperature-controlled organ bath chambers containing 5 milliliter (ml) of Krebs solution bubbled with 95% O_2_/5% CO_2_ at 37°C. One end of each uterine strip was attached to a fixed glass hook at the bottom of the organ bath chamber and the other end was hooked to an isometric force transducer (6240 biological treatment systems). Changes in isometric contraction were recorded.

### Isometric contraction measurement

Preliminary tension-contraction curves using rat uterine strips under increasing basal tension 0.5, 1, 2, 3, 4, 5, 6, 7, and 8 g, maintained under each basal tension for 30 min, then stimulated with 96 mM KCl, demonstrated maximal contraction at 2 g basal tension, no significant change in contraction at 2 to 7 g basal tension, and a significant decrease in contraction at high 8 g stretch [1]. Therefore, uterine strips of pregnant rats were stretched under control 2 g basal tension and allowed to equilibrate for 1 hour (h) in Krebs solution. Uterine strips were stimulated with 96 mM KCl to obtain a maximal response. Each control KCl contraction was followed by three washes in Krebs, 10 min each. KCl contraction was repeated twice. Preliminary oxytocin concentration-response curves in uterine strips suggested that 10^–7^ M produced the maximal uterine contraction, and therefore this oxytocin concentration was used in all subsequent experiments. To determine the effects of progesterone, the uterine strip in each organ bath chamber was incubated in increasing concentrations of progesterone (10^–7^ to 10^–5^ M, Sigma-Aldrich) for 1 h then stimulated with oxytocin 10^–7^ M for 1 h. To determine whether TREK-1 is involved in the changes in uterine contraction induced by progesterone, contraction experiments were performed in the presence of TREK-1 inhibitor L-methionine (1 mM) (Sigma-Aldrich) or activator arachidonic acid (10^–5^ M) (Sigma-Aldrich). Different uterine strips showed variable baseline contractile activity. The uterine strips were assigned to control and treatment groups at random, and therefore the basal contractile activity is less likely to affect the cumulative data from different uterine strips from different animals. Also, the uterine contractile response showed both a steady-state contraction and an additional phasic contraction. Because the phasic contractile response showed significant variability in frequency and amplitude, the area under the curve (AUC, R1640 data analysis system) representing the total uterine contraction (steady-state + phasic) was measured. Oxytocin AUC was measured at 20 min intervals from oxytocin application, and normalized to the tissue weight in gram (g). In some experiments, the uterine strip was equilibrated under 8 g stretch to test the effects of uterine stretch on contractile function and the progesterone-induced effects on uterine contraction.

### Real-time quantitative PCR detecting system, qPCR analysis

Uterine strips were frozen in liquid nitrogen and stored at –80°C. Total tissue RNA was isolated from the uterine strips using TRIzol (Pufei, ShangHai). The RNA concentration of the sample was tested by the Nanodrop 2000/2000C spectrophotometer. Total RNA (2 µg) was used for RT to synthesize single-strand complimentary DNA (cDNA) in a final 25 µl reaction mixture according to the instructions of the First-Strand cDNA Synthesis Kit (Beyotime Biotechnology). RT product contained 1 µl cDNA dilution (1:20 for TREK-1 and GAPDH) was applied to 20 µl of RT-PCR reaction mixture.

The PCR was performed with a real-time RT-PCR machine (model MX3000p, Agilent), published oligonucleotide primers for TREK-1 (Shanghai GeneChem Co., Ltd), and SYBR Master Mixture, which employs the fluorescein compound SYBR-Green for amplicon detection (TAKARA). GAPDH primer was used as an internal control to normalize the results in the RT-PCR reaction. The following primers were used: TREK-1: Forward, 5′-CCAGGAACTGACTCCGTGTAG-3′; reverse,5′-GGTGTCAGACCGTTCAGATAGA-3′; GAPDH: Forward, 5′-TGACTTCAACAGCGACACCCA-3′; reverse, 5′-CACCCTGTTGCTGTAGCCAAA-3′

After initial denaturation at 95°C for 10 min, the reactions were taken though 40 cycles: 30 s at 95°C, 45 s at 59°C and 30 s at 72°C, followed by 1 min of final extension at 95°C. The optimum number of cycles for the appropriate primer was determined by the expression level of the target gene. This PCR protocol provided optimized conditions and linear range for a proportional relationship between input RNA and the cycle’s readout.

The amount of mRNA relative expression in each sample was measured by comparison of cycle thresholds with the housekeeping gene GAPDH.

### Western blot analysis

The uterine strips were homogenized in RIPA lysis buffer (Beyotime Biotechnology) and centrifuged at 10000 g for 5 min. After centrifugation, the protein concentration in the supernatant was measured using BCA protein assay kit (Beyotime Biotechnology). Samples were subjected to electrophoresis on 10% SDS polyacrylamide gel, then protein was transferred electrophoretically from gels to PVDF membranes (Millipore). The membranes were blocked with 5% nonfat dry milk in PBS-Tween buffer for 1 h, and then incubated overnight at 4°C with antibody solution containing TREK-1 (1:1000) rabbit polyclonal antibody (Sigma). GAPDH was used as an internal control and detected by a monoclonal antibody (1:500000, Sigma). After washing in PBS-Tween, the PVDF membranes were incubated in horseradish peroxidase conjugated secondary antibody (1:5000) for 1.5 h. The membrane blots were washed with PBS-Tween and visualized with enhanced chemiluminescence (ECL). The reactive band corresponding to TREK-1 was analyzed by optical densitometry and ImageJ software (National Institutes of Health). The densitometry value represented the pixel intensity normalized to GAPDH to correct for loading.

### Solution and drugs

Normal Krebs solution contained (in mM): 120 NaCl, 5.9 KCl, 25 NaHCO_3_, 1.2 NaH_2_PO_4_, 2.5 CaCl_2_, 1.2 MgCl_2_, 11.5 dextrose. Krebs solution was bubbled with 95% O_2_/5% CO_2_ at 37°C for 30 min at an adjusted pH 7.4. 96 mM KCl was prepared as normal Krebs but with equimolar substitution of NaCl with KCl. Stock solution of oxytocin (10^–4^ M, Shanghai Hefeng Pharmaceutical Company) was prepared in distilled water. Experimental solution of TREK-1 channel inhibitor L-methionine (10^–3^ M, Sigma) was prepared in Krebs solution. Stock solution of TREK-1 channel activator arachidonic acid (10^–2^ M, Sigma) was prepared in anhydrous ethanol. The final concentration of ethanol was less than 0.1% and had no effect on uterine contraction.

### Statistical analysis

Cumulative date from uterine strips of different rats were analyzed and presented as means ± SEM, with “n” representing the number of rats. Data were analyzed using ANOVA with multiple classification criteria [tissue treatment (treated with progesterone, L-methionine or arachidonic acid vs nontreated control tissues), drug dose, and tissue stretch (control 2 g basal tension vs 8 g stretch)]. When a statistical difference was observed, the data were further analyzed using Student-Newman-Keuls *post-hoc* test for multiple comparisons. Student’s unpaired *t*-test was used for comparison of two means. Differences were considered statistically significant if *P* < 0.05.

## Abbreviations

KCl: potassium chloride
AUC: area under the curve
